# The psychosocial needs of adolescent and young adult kidney transplant recipients, and associated interventions: a scoping review

**DOI:** 10.1186/s40359-022-00893-7

**Published:** 2022-07-29

**Authors:** Fina Wurm, Clare McKeaveney, Michael Corr, Anna Wilson, Helen Noble

**Affiliations:** 1 Medical Biology Centre, School of Nursing and Midwifery, Queen’s University Belfast, 97 Lisburn Road, Belfast, BT9 7BL Northern Ireland, UK; 2 Centre of Public Health, Queen’s University Belfast, Belfast, Northern Ireland, UK

**Keywords:** Renal transplantation, Paediatric psychology, Health psychology, Renal replacement therapy, Adolescent and young adult

## Abstract

**Background:**

Renal transplantation is considered the gold standard treatment for end-stage kidney disease. Adolescent and young adult kidney transplant recipients have the highest rate of graft loss amongst transplanted patients. It is largely accepted this is due to psychosocial and behavioural difficulties, which impact adherence to prescribed therapies. This phenomenon is not isolated to a particular healthcare system having been observed in multiple countries across different continents. It is a global issue of concern. We sought to review the psychosocial needs of these patients, and the interventions designed to meet these needs.

**Methods:**

A scoping review was conducted based on Arksey and O’Malley’s six-stage framework. Eligibility criteria included primary studies of any type that investigated the psychosocial needs of adolescent and/or young adult renal transplant recipients or studies which examined interventions designed to address these needs. Search strategies were developed and conducted on PsycINFO, PubMed, Embase, and CINAHL. Articles meeting the inclusion criteria were critically reviewed using a descriptive-analytical narrative method.

**Results:**

Thirty-nine studies met our inclusion criteria, 30 of which related to psychosocial needs, and the remainder examined psychosocial interventions. Four main themes were derived from our analysis of psychosocial needs literature, as follows: the need for (1) emotional support, (2) acceptance, (3) direction, (4) equality in healthcare. 2 main themes emerged from analysis of psychosocial intervention literature, namely: psychosocial (1) capability, (2) assessment. Despite the evidence that graft health is strongly associated with psychosocial wellbeing, findings revealed a significant lack of literature investigating how best to meet psychosocial needs. Trends were observed amongst intervention studies, namely interventions of novel and non-evidenced based design, with the aim of improving medication adherence through organisational strategies and education. However, literature regarding psychosocial needs showed non-adherence to therapies was not simply a result of disorganisation or lack of understanding, but rather, was founded on a recipient’s idiosyncratic relationship with their prescribed therapies e.g., psychological, social or financial reasons for non-adherence.

**Conclusions:**

Future research should be directed at investigating the efficacy of evidence-based interventions that empower the individual patient to overcome their specific barrier to an optimal relationship with their therapies.

## Background

End-Stage Renal Disease (ERSD) is a condition that is rare amongst adolescent^1^ and young adults^2^ in the United Kingdom [1]. Patients with ESRD require Renal Replacement Therapy (RRT). Transplantation is one form of RRT; other forms include of haemodialysis (HD) and peritoneal dialysis (PD), the aim of which is to substitute the normal blood-filtering function of the kidneys [2]. Transplantation is often referred to as the gold standard treatment, as compared to dialysis, it improves life expectancy, reduces associated comorbidities, and improves the recipient’s quality of life [3]. Transplantation offers, particularly for younger patients, better opportunity for integration into ‘normal’ life, as when it is successful, it eradicates dependency on dialysis which is an intensive time-consuming medical intervention [4]. Dialysis, accordingly, inhibits the young patient’s ability to sustain ‘normality’ e.g., maintain education, hobbies, and friendships, as it requires the patient to be connected and constricted to a dialysis machine for approximately three sessions of 4 h a week, typically carried out in hospital [5]. During ETRD the patient experiences extreme fatigue and inability to concentrate, in addition to other debilitating symptoms which create further barriers to psychosocial functioning e.g., compromised immune function [6]. Adolescent and young adult patients report clinically significant symptoms of depression, anxiety, and stress throughout their time on dialysis [7] which can last beyond transplantation [8]. Transplantation offers the young patient the best opportunity for ‘normal’ life that is free from intensive medical intervention like dialysis [9], thus it is imperative transplantation is successful to avoid having to return to it.

A serious potential difficulty that may arise post-transplant is graft loss. Graft loss describes the loss of kidney transplant function and when it occurs, the patient must return to dialysis. Mortality rate increases, and the risk of developing associated morbidities including cardiovascular problems and infection increases [10].

A return to dialysis is also associated with higher economic costs as sustaining dialysis is expensive with the average cost being £30,800 per patient per year [11, 12]. For each year that a patient has a functioning kidney transplant instead of returning to dialysis, the cost benefit to the NHS is £24,100 per year, equating to approximately £60million in saving per year [11]. Thus, ensuring successful transplantation is important not only on an individual basis, but also nationally, as it impacts the economic system [12].

Adolescent and young adult transplant recipients have the highest rate of graft loss amongst transplant recipients [13]. This is a global phenomenon, having been observed in multiple countries, including Canada [14], the United Kingdom [15], America [13] Germany and Austria [16], New Zealand and Australia [17]. There have been many hypothesised reasons for this. Some have suggested that this population has higher immunological activity and others have suggested they have higher metabolism of immunosuppression medications [18]. However, most transplant nephrologists and subsequently the literature believe the high rates of transplant graft loss in this group is related to behavioural challenges and lack of adherence to prescribed therapies [17]. Meaning, it is psychosocial rather than biological factors impacting transplant outcome [17]. Taken together, this issue is not isolated to a particular healthcare system, thus there is a global need to understand the psychosocial needs of kidney transplant recipients, and the interventions available to them.

Despite the importance for adolescent and young adult wellbeing and subsequent graft success, a body of literature concerning psychosocial needs and available interventions does not exist within nephrology literature. Reviews examining psychosocial aspects of paediatric patient experiences of chronic illnesses have been conducted in oncology [19], epilepsy [20], diabetes [21], asthma [22], irritable bowel syndrome and recurrent abdominal pain [23] and appearance altering conditions [24]. The purpose of this scoping review is to provide an overview of research evidence in nephrology.

### Aims and Research Questions

This scoping review aimed to:

1. Investigate the psychosocial needs of adolescent and young adult renal transplant recipients
2. Review psychosocial interventions that are available to help address these patients’ psychosocial needs

The following research questions were developed:

1. What is known about the psychosocial needs of adolescent and young adult renal transplant recipients?
2. What psychosocial interventions are available to help address these patients’ psychosocial needs?

## Materials and methods

The review was guided by the methodological framework of Arksey and O’Malley [25] later developed by Levac and Colquhoun [26]. This framework consists of five key stages, where stage six is considered optional [26]:

1. Identifying the research question(s)
2. Identifying relevant studies
3. Study selection as per the inclusion protocol
4. Charting the data. Relevant information was extracted from the literature and documented on an Excel spreadsheet.
5. Collating, summarising and reporting the results in tables and charts according to key themes, with an analytical summary of the findings
6. Translating the knowledge to key stakeholders through consultation [26].

### Identifying the research question

This scoping review was developed to describe the nature, number and scope of published research articles examining the psychosocial needs of young kidney transplant recipients in addition to articles examining interventions designed to help address this need.

### Identifying relevant studies

A systematic literature search of the databases, PsycINFO, PubMed, Embase, and CINAHL was conducted of all published articlesusing anelectronic search strategy thatincluded MeSH headings, key words and their derivatives (Tables 1 and 2). The terms and the search criteria were developed and tested with the Queens University School of Nursing and Midwifery’s librarian. All articles were downloaded into Endnote and duplicates were removed.

**Table 1:** Search strategy: psychosocial needs

	Search term	No. of publications found
1	Young adult.mp. or young adult	448,481
2	Adolescent/	1,606,836
3	Kidney transplantation.mp. or kidney transplantation/	132,736
4	Renal transplant.mp. or kidney graft/	69,375
5	Psychosocial.mp. or psychosocial care/	162,722
6	Interventions.mp. or intervention study/	682,188
7	Therapy/ or therapy.mp	8,321,259
8	1 or 2	1,902,851
9	3 or 4	163,304
10	6 or 7	8,759,035
11	5 and 8 and 9 and 10	303

**Table 2:** Search strategy: psychosocial interventions

	Search term	No. of publications found
1	Young adult.mp. or young adult	1,570,903
2	Adolescent/	3,713,104
3	Kidney transplantation.mp. or kidney transplantation/	238,376
4	Renal transplant.mp. or kidney graft/	172,641
5	Psychosocial.mp. or psychosocial care/	390,509
6	Support.mp	12,180,116
7	Needs.mp	1,133,128
8	1 Or 2	4,623,974
9	3 or 4	272,794
10	6 or 7	13,279,508
11	5 and 8 and 9 and 10	77

### Study selection

The titles and abstracts of all identified studies were screened by the research team using an inclusion and exclusion criteria (Table 3). One researcher independently applied the inclusion criteria to each abstract to determine whether to review the full text. Of all the eligible abstracts, half were assigned to another reviewer, and the other half were assigned to a third reviewer. The review team met to compare screened abstracts and any differences were resolved through consultation. To identify any further studies that had been missed in the electronic searches, the reference lists of eligible studies were also reviewed.

**Table 3:** Inclusion and exclusion criteria

Abstract	*Inclusion*
	Renal transplant recipients (post-transplant) aged 10–30
	Empirical peer-reviewed research
	Language: English
	Psychological and/or social aspect of patient’s experience explored with/without other topics
	Intervention studied is targeted at psychological and/or social wellbeing with/without other topics
	*Exclusion*
	Other organ transplant recipients
Full text	*Inclusion*
	Relevant data must be extractable if studied with other: organ transplant recipients, age groups or topics
	Average age of participants is within ages 10–30
	Clearly states psychological and/or social need of participant
	Clearly states intervention (rationale, design, outcome) targeted at psychological and/or social wellbeing of participant

### Data extraction

After reading the full texts of each study to be included in the review, one researcher independently extracted the following data: author(s), year of publication, study design, study location, participant characteristics, psychosocial needs and how they were measured, psychosocial interventions and their objectives for implementation, and the main findings. Data was initially extracted from the first 5 studies and compared by two reviewers to ensure consistency.

### Collating and summarising the data

After title and abstract screening, 145 articles for psychosocial needs and 37 articles for psychosocial interventions proceeded to full text review.

Following full text review 30 articles in total were accepted for inclusion for psychosocial needs and 9 articles were included for inclusion for psychosocial interventions.

The data from the included studies was collated to provide both a descriptive and numerical summary of the findings and to answer the research questions.

## Findings

Removing duplicates and screening abstracts and full texts resulted in the inclusion of 30 published articles related to psychosocial needs. This included 23 research studies, 2 systematic reviews and 5 literature reviews. The quantitative research studies included 2 randomised control trials, 2 quasi-experimental studies, 10 cross-sectional surveys and 5 cohort studies. The qualitative research studies included 2 phenomenological studies. A further 2 research studies used a combination of quantitative and qualitative approaches.

Removing duplicates and screening abstracts and full texts resulted in the inclusion of 9 published articles for psychosocial interventions. This consisted of 8 quantitative studies [3 non-randomised control trials, 1 service evaluation report and 4 randomised control trials]. The qualitative research included 1 phenomenological study.

Four key themes were identified in relation to psychosocial needs: (a) the need for emotional support; (b) the need for acceptance; (c) the need for direction; (d) the need for equality in healthcare.

Two key themes were identified regarding psychosocial interventions: (a) psychosocial capability; (b) psychosocial assessment.

### Psychosocial needs

#### The need for emotional support

While there is an association between receiving a transplant and improved quality of life, findings revealed that recipients can experience poor mental health after transplantation [27–36]. Anxiety disorders were the most prevalent reported mental health disorders in the literature [27–33]. One study reported symptoms in keeping with Post-Traumatic Stress Disorder (PTSD) including nightmares, irritability, and insomnia, and noted the younger the patient the more severe the symptoms [27]. Furthermore, they found the ritual of taking medication could trigger PTSD symptoms [27]. Generalised Anxiety Disorder (GAD) was reported to be the most prevalent mood disorder amongst Turkish adolescent patients [28]. Worry (a characteristic of the disorder) was experienced by other adolescent and young adult patients regarding the uncertainty of the future [30, 35], their appearance [31, 32] and familial adjustment immediately following transplant [34]. Re-entering school was a further trigger for other emotions inclusive but not limited to anxiety [30, 31]. Some struggled with their academic regression and felt shame, guilt and blame for their drop in academic scores [30]. They felt personally responsible for not coping well enough and believed they had allowed their illness to impact the academic achievements. Others felt social isolation on return to school and reported the cause to be bullying and name calling regarding physical appearance [30]. Another study found physical appearance to be a significant positive predictor of perceived ability to adjust back in education, meaning adolescent patients felt less able to cope with the prospect of school if they experienced negative body image [31]. A further trigger for negative emotions was sexual functioning [33, 35–37]. Sexual functioning and delayed psychosexual puberty in young adolescents were found to cause social isolation, although whether this was due to self-isolation or bullying was not stated [35]. Interpersonal stress was observed when there was an unequal desire toward sex between a recipient and their romantic partner [34, 37], typically due to transplant recipients reporting health-related physical symptoms e.g., fatigue and nausea, impacting their libido [37]. Other recipients experienced stress with partners due to feeling that intimacy was of less importance to them, than their partner [37]. Male young adult recipients reported unhappiness due to experiencing poorer orgasm and less desire toward sex posttransplant [36]. As no erectile dysfunction was reported, the authors suggest psychological symptoms, likely of depression, to be impacting sexual functioning rather than biological causes [36].

#### The need for acceptance

While the relationship between acceptance of illness and psychosocial wellbeing posttransplant has not been studied, findings suggested a causal relationship between these variables [27, 30, 38–41]. Adjustment to life posttransplant appeared to be aided when patients accepted their illness [30, 38, 39]. Young recipients adjusted to taking medication by accepting it as part of their new daily routine [30]. Adolescent recipients showed positive social adjustment by accepting their illness, as it allowed them to build an identity separate to it [30]. Accepting the illness as part of their identity, motivated younger patients to connect with patients of the same age [38, 39]. Doing so fulfilled their sense of belonging [39]. Adult recipients that accepted their illness and its impact on their futures, were able to set realistic goals, which gave them a sense of purpose and fulfilment [38]. Furthermore, accepting their illness as part of the narrative of their lives promoted self-reflection, resilience, and healthy coping strategies [39]. Adaptive coping involved volunteering with other renal patients, working in healthcare, donating money to healthcare charities, seeking medical advice from professionals when needed, speaking with loved ones when needing support, and using downward social comparison (comparing oneself to those one perceives as being worse off) to put their health-related problems into perspective [39]. Many studies suggested a causal relationship between denial of illness and maladaptive behaviours [27, 39–41]. Denying they had a chronic illness led to higher non-attendance at hospital and worse adherence to medical therapies in adolescent patients [27]. They also expressed disinterest in connecting with other renal patients [40]. Adult recipients longed to temporarily deny their negative emotions using substance misuse to cope, while others found video gaming encouraged positive escapism [39]. Coping mechanisms of young adolescent patients were shown in one study to also be affected by denial, in that patients with the mildest physical symptoms demonstrated the worst coping strategies [41]. The authors hypothesised potentially they considered themselves not sick enough, to need to acquire coping skills [41].

Acceptance by other people was a significant consideration for the adolescent and young adult transplant recipients [30, 31, 33, 37, 42]. Young adult patients were concerned about finding a romantic partner who would accept their health problems including such issues as decreased fertility, potential of hereditary disease and decreased life expectancy [30, 37]. A prevalent theme in the literature was the need to physically look like peers to be accepted socially [30, 31, 33, 42]. Patients of all ages reported physical alterations of transplant (scars, weight gain, swelling and stunted growth) acting as painful reminders of being different from others [30]. One study found adolescents worried daily about their weight, shape, and size in comparison to that of their healthy peers [31]. Adolescent patients reported being more concerned about the cosmetic consequences of their medication, than the health-related side-effects associated with it [33]. Another study found adolescent patients with negative body image were less likely to adhere to taking posttransplant medications [42]. Therefore, medication may be negatively perceived for the young patient, due to the relationship between physical alterations and social acceptance.

#### The need for direction

The literature showed adolescent patients had better psychosocial and physical outcomes when parents offered direction [27, 33, 38]. Direction was given in the form of demonstrating healthy coping strategies including stress management [43] and emotional expression [33]] and was associated with better psychosocial adjustment and fewer hospitalisations [33, 43]. Findings showed higher rates of adherence to medication when living with parents rather than living alone, suggesting parents offered direction in the form of medication management [42]. One study found older adolescent patients were supported in managing their medication unsupervised after a period of supervision [38]. Potentially this approach mitigates the likelihood of adolescent patients refusing to take medication in the attempt to regain independence and control, as a different study observed [27]. Furthermore, offering initial guidance prior to unsupervised management lead to successful medication adherence, whereas adolescent patients that were unsupervised in medication management immediately following transplant showed poor adherence rates [27, 44, 45]. Data comparing social demographics of recipients and healthy peers highlight the need for the young patient to be supported navigating life as an independent adult following transplantation [37, 42]. Compared to healthy peers, recipients were more likely to lead healthy lifestyles demonstrated in lower alcohol intake, lower rates of smoking and lower engagement with crime [42]. Despite appearing to follow a similar psychosocial trajectory as healthy adolescent peers, as seen by the similar age to first have sex and try alcohol and cannabis [42] they were more likely to be living with parents, more likely to be unemployed and living without an income, less likely to be romantic relationships and less likely to have friends [37]. The disparity in reaching adult milestones elucidates a potential difficulty navigating life independently as a young adult.

Recipients desired direction from healthcare staff in the form of education related to issues such as the technical, medical and experience aspects of the surgery, the effect of transplant on physical appearance and the effect of drugs and alcohol on physical wellbeing [46]. Recipients need education from healthcare providers on the relationship between graft loss and medication adherence [27, 38]. One study found understanding the importance of medication lead to higher levels of motivation to take it [38]. Another found that 76% of adolescent patients did not know what their medication was for, accordingly most of these patients did not take medication regularly [27]. Knowledge deficits were significantly correlated with medication adherence in another study, with a large population of over 400 participants [42], adding to the validity of this phenomenon. Findings of one study suggested patients would benefit from healthcare staff managing their expectations of health-related quality of life posttransplant [30]. It found that many young people felt they had put their life on hold until transplantation, only to discover unexpected different challenges awaiting them afterwards [30]. Thus, goals set prior to transplant were unrealistic and unattainable [30].

#### The need for equality in healthcare

Literature pertaining to the psychosocial needs of paediatric patients predominantly study middle class Socioeconomic Status (SES), Caucasian populations. A small body of literature elucidates the unique psychosocial needs of minority patients, of low SES and predominantly of Black, Asian and Minority Ethnic (BAME) race and culture. ‘Findings from these studies demonstrated that recipients in poverty experience significant adversity’ [47–55]. A prominent cause is lack of socialised healthcare systems. Studies conducted in America and India where healthcare is privatised, have shown inability to afford medications to be a significant predictor of graft loss [37, 47, 48]. Insurance can also affect the patient’s likelihood of getting a transplant when policy does not cover the cost of their donor parent or guardian’s care [49]. Poverty is a universal issue that decreases the paediatric recipient’s likelihood of recovery [41, 47, 50–55]. Reasons for this have been elucidated by studies examining the relationship between low SES demographics and transplant outcome [47, 49–53]. The following social factors were significantly associated with adverse outcomes: having a low-income zip code predicted graft loss [47]; being a black African American young man made one the highest risk group for posttransplant cannabis addiction, and graft loss [50]; Single parent households predicted worse emotional adjustment [51]; mothers’ education status predicted IQ scores posttransplant [52]; fatherless households predicted worse medication adherence [53]; poor communication due to language barriers between a patient, their family and medical team was associated with worse adherence to medications [47]; undocumented children often had longer waiting times for specialist treatment due to the added legal and financial complexity involved in their care [49]. Racism is another problem experienced by BAME patients disproportionately [54–56]. Studies [54–56] highlighted the following: BAME patients’ outcomes on a global scale across America, Australia, and in 36 European Countries have been negatively impacted by racism; racial bias has contributed to BAME patients being less likely to be chosen for pre-emptive transplantation and to be considered eligible for waitlisting; studying the impact of racism on outcomes alone is a hugely complex task as confounding variables such as medical factors and psychosocial determinants for health are difficult to accurately control for.

### Psychosocial interventions

The body of literature examining interventions addressed to meet psychosocial needs was significantly smaller than the body of literature identifying what these needs are. The findings revealed two themes: psychosocial capability and psychosocial assessment.

#### Psychosocial capability

Most psychosocial interventions were designed to improve the adolescent or young adult’s capability to cope with medical regimes posttransplant. Two studies sought to enhance the recipient’s knowledge of medications through a computerised series of modules [57, 58]. One found an increase in self-reported knowledge postintervention [57], while the other reported no difference between groups [58]. Capability was further improved by interventions designed to support the young recipient to practically manage their fluid intake [59] and medications [60, 61]. One intervention utilised electronic pill boxes with personalised medication reminders, in addition to receiving problem solving coaching every 3 months [60]. The authors found a significant improvement in medication adherence following the intervention, however, did not measure which variable (pill box, reminders, or coaching) was most influential for positive behaviour change [60]. Self-management of fluid intake was improved through a simple strategy which employed an interactive electronic water bottle [59]. Recipients were more likely to reach their targeted fluid levels each day and reported enjoying the experience of using the bottle [59]. One study sought to improve self-management of medication taking through a family approach, which consisted of parents and recipients breaking tasks into smaller components, and parents handing over control of medication management in a gradual manner [61]. While the literature suggests capability to follow medical regimes (by increasing knowledge and supporting self-management) can be improved through intervention, the long-term effect on health-related behaviour change is unknown given that efficacy was measured immediately after intervention.

Psychosocial interventions were designed to improve the recipient’s capability with coping during the transition process from paediatric to adult services [39, 62, 63]. Interventions were often complex in nature with multiple variables being studied at the same time, thus it was unclear which intervention accounted for which outcome [1]. For example, one study found working with a specialised nurse and social worker reduced graft failure during the transition process [62], while another found working with a social worker and transplant co-ordinator had no effect on graft health [63]. Furthermore, some studies did not specify what kind of support was given to the recipient from the staff implemented to support them during this process e.g., the topics discussed between youth worker and recipient [62, 63], making it difficult to understand how to implement successful interventions. Of those that did elucidate processes with more specificity, one study showed increased capability as observed through improved self-reported mental health, increased adherence to medications, and enhanced feelings of resilience for those in the intervention groups [39]. Interventions consisted of learning practical skills including organisational and goal setting [39] and having discussions regarding psychosocial issues [39]. However, the theoretical approaches underpinning the specific ways skills were taught or discussions were facilitated were not stated. Taken together, implementing these interventions is both difficult given their ambiguity and therefore, potentially unethical, considering the evidence-base underpinning specific therapeutic processes is difficult to infer [39, 62, 63].

#### Psychosocial assessment

Three of the selected studies examined interventions designed to assess psychosocial status of adolescent and young adult transplant recipients [64–66]. One study implemented an annual review with a clinical psychologist, with the aim of screening for psychosocial distress [64]. The study found that through an annual review, patients with symptoms of psychosocial issues e.g., depression or anxiety, could be identified and referred to appropriate services for treatment. However, there was no follow-up measurement to see if referred patients sought the treatment they were recommended. The study mentions without much elaboration, that two thirds of patients without psychosocial difficulties, had previously seen a clinical psychologist for treatment. This suggests that psychological therapy with a clinical psychologist can have lasting effects in eradicating psychosocial distress for recipients and may be an effective intervention to address psychosocial needs. A second study sought to examine the efficacy of using an arts-based intervention to assess psychosocial wellbeing [66]. Recipients were asked to prepare five photographs which they felt reflected their experiences posttransplant. The authors noted that data collected this way was richer than data collected through standard interview and thought it enhanced self-reflection, however controlled comparative groups were not studied to confirm this. The third study assessed symptoms of depression and posttraumatic stress through art, examining the effectiveness of the Formal Elements of Art Therapy Scale (FEATS) [65]. The authors concluded that FEATS was at that point, an invalid measurement of psychosocial distress. However, they suggested engaging with art could have benefits to renal recipients based on the developmental appropriateness of the medium, and the observed ease with which participants of all ages engaged with it during the study. The authors suggested future research could build on this, exploring the effect of engaging with art, or other arts-based interventions (music and dance) on psychosocial wellbeing posttransplant.

## Discussion

This scoping review sought to explore what is known about the psychosocial needs of adolescent and young adult kidney transplant recipients, and identify interventions designed to address these needs. Findings elucidated numerous psychosocial needs; (a) the need for emotional support; (b) the need for acceptance; (c) the need for direction; (d) the need for equality in healthcare; the majority unmet by lack of or methodological limited interventions. Analysis of psychosocial intervention studies elucidated a trend amongst the literature, which was a desire to change behaviour [57–61]. The predominant objective of psychosocial interventions was behaviour change to increase medication adherence by improving the recipient’s capability of managing their medication. Targeting adherence through self-management strategies and increased medication related knowledge implies the predominant psychosocial need of these recipients is one of organisation or education, however, the literature detailing psychosocial needs strongly suggests non-adherence is caused by a recipient’s idiosyncratic appraisal of the medication. For example, a recipient may see non-adherence as a means of preventing undesirable cosmetic features [30], as an opportunity to regain independence and power [27] or as the only option available to them because they are unable to afford medications 48). Thus, an important gap in the literature has been identified; interventions that aim to problem solve non-adherence with a client-centred approach, considering the unique demographics of the patient and their specific barriers to non-adherence. Secondly, there is an urgent need to investigate pre-existing evidence-based therapies rather than designing novel ideas as the current literature has focused on [57–61]. Cognitive Behavioural Therapy (CBT), a type of psychological therapy, is suitable across age-groups and neurodevelopmental ability, and is effective in short periods of time, making it cost effective [67]. Taking a cognitive behavioural approach has been shown to significantly improve medication adherence in other paediatric populations in as quickly as 8 weeks [67]. Another evidence-based tailorable intervention is Dialectical Behavioural Therapy (DBT), which has already been showed to significantly increase adherence to medical therapies in adolescents on dialysis after 9 weeks [68].

Literature detailing psychosocial needs suggested a causal relationship between psychosocial wellbeing, successful adjustment, and optimal medication adherence. This relationship highlights the potential to improve adherence by proxy of improving other aspects of psychosocial functioning. Doing so could also reap benefits on a national level as patients reintegrate as functioning, capable, members of society. For example, non-adherence to medication is associated with high depression scores and unemployment [69]. Interventions designed to support recipients navigating employment posttransplant while receiving mental health treatment, would benefit them socially, psychologically, and financially, as well as benefiting the economic system as they join the workforce. A large gap in the literature prevails regarding interventions designed to address psychosocial needs with the aim of improving psychosocial functioning in addition to, or instead of adherence to prescribed therapies. Furthermore, given that only five of 42 studies examining psychosocial needs used qualitative methodology to ask recipients what their perceived needs were [28, 33, 37, 39, 50] and no studies quantitatively or qualitatively asked recipients how to meet these needs, a gap prevails identifying the young recipient’s own opinion of how best to help them achieve psychosocial wellbeing in their unique context e.g., their healthcare system, race, SES, age, culture, or sex.

### Future research

As graft health is significantly determined by psychosocial wellbeing, a biopsychosocial approach is needed to overcome a biopsychosocial problem. Additionally, future research should be conducted in a manner that promotes diversity, inclusivity, and social inclusion by making a concerted effort to study the psychosocial experiences of those most at risk namely patients from minority groups i.e., socially disadvantaged patients and/or of BAME backgrounds. Associated subjects that have yet to be studied comprehensively e.g., where most participants are from minority backgrounds making findings generalisable to them or where the focus is exclusively on this population include transplanted renal patients: with advanced cognitive difficulties e.g., learning disabilities’; from minority backgrounds e.g., Irish or English Traveller; or with complex co-morbidity e.g., deafness, blindness or disorders like addiction or psychosis.

Suggestions for addressing psychosocial needs through future research consist of mixed methods, short-term approaches and long-term approaches that seek to involve the pre-existed evidence-based therapies and design of novel psychosocial interventions. For example, one could investigate the efficacy of a short-term CBT treatment program for adolescent transplanted patients experiencing generalised anxiety disorder or depression. Furthermore, discourse on how best to meet the needs of these recipients could be conducted through multidisciplinary teams, rather than those founded predominantly in the medical model as is currently happening.

### Limitations

It is feasible that despite an extensive search of multiple databases, some relevant papers may have been missed. Language barriers may have caused relevant papers in languages other than English to be dismissed. Not all abstracts and full texts were screened by two reviewers, however, the vast majority were. There was also no quality appraisal or meta-analysis of the included studies undertaken, but this is not deemed to be part of the scoping review process.

## Conclusion

This is the first scoping review of psychosocial needs and psychosocial interventions for adolescent and young adult kidney transplant recipients. Findings from this review showed the potential adverse psychosocial experiences associated with renal transplantation in this patient population. It has elucidated to an unmet need for psychosocial care before and long after the recipient’s transplant. To sustain graft health, psychosocial wellbeing is imperative, however, there is a significant lack of literature examining interventions designed to address these needs. Of the interventions designed trends were observed, namely a focus on improving self-management of medication through organisational skills and interventions that were novel but non-evidenced based. Future research needs to consider that non-adherence to therapies is not just a result of disorganisation, but rather, is founded on a recipient’s unique relationship with their medication e.g., psychological, social or financial reasons for non-adherence. Therefore, future research must investigate the efficacy of evidence-based interventions that tailor to the individual, increase quality and quantity of involvement, empowering them to overcome their specific barrier to an optimal relationship with their medication. Future research should also involve recipients more than has been done in the past, by simply asking them what they believe their needs are, and what they think would be of use to meet them. Responses should be collected across diverse demographics and collated systematically for future research regarding intervention design. Finally, a multidisciplinary approach is needed as expertise across healthcare fields must come together to solve a uniquely biopsychosocial global problem in a way that promotes diversity and social inclusion.

## Data Availability

The datasets used and/or analysed during the current study are available from the corresponding author on reasonable request.

## Footnotes

1 Defined by the World Health Organisation as anyone between the ages of 10 and 19.

2 Defined as anyone under the age of 30.
